# Biochemical Characterization of Novel GH6 Endoglucanase from *Myxococcus* sp. B6-1 and Its Effects on Agricultural Straws Saccharification

**DOI:** 10.3390/foods12132517

**Published:** 2023-06-28

**Authors:** Zhen Huang, Guorong Ni, Longhua Dai, Weiqi Zhang, Siting Feng, Fei Wang

**Affiliations:** 1 College of Bioscience and Bioengineering, Jiangxi Agricultural University, Nanchang 330045, China; huangzhen_918@163.com (Z.H.); 18770052522@163.com (L.D.); zwq13707923026@163.com (W.Z.); 17807025798@163.com (S.F.); 2 College of Land Resources and Environment, Jiangxi Agriculture University, Nanchang 330045, China; ngr314@163.com

**Keywords:** endo-β-1,4-glucanase, *Escherichia coli*, expression, characterization, oligosaccharide, saccharification

## Abstract

Cellulase has been widely used in many industrial fields, such as feed and food industry, because it can hydrolyze cellulose to oligosaccharides with a lower degree of polymerization. Endo-β-1,4-glucanase is a critical speed-limiting cellulase in the saccharification process. In this study, endo-β-1,4-glucanase gene (*CelA257*) from *Myxococcus* sp. B6-1 was cloned and expressed in *Escherichia coli*. CelA257 contained carbohydrate-binding module (CBM) 4-9 and glycosyl hydrolase (GH) family 6 domain that shares 54.7% identity with endoglucanase from *Streptomyces halstedii*. The recombinant enzyme exhibited optimal activity at pH 6.5 and 50 °C and was stable over a broad pH (6–9.5) range and temperature < 50 °C. CelA257 exhibited broad substrate specificity to barley β-glucan, lichenin, CMC, chitosan, laminarin, avicel, and phosphoric acid swollen cellulose (PASC). CelA257 degraded both cellotetrose (G_4_) and cellppentaose (G_5_) to cellobiose (G_2_) and cellotriose (G_3_). Adding CelA257 increased the release of reducing sugars in crop straw powers, including wheat straw (0.18 mg/mL), rape straw (0.42 mg/mL), rice straw (0.16 mg/mL), peanut straw (0.16 mg/mL), and corn straw (0.61 mg/mL). This study provides a potential additive in biomass saccharification applications.

## 1. Introduction

Cellulose is the principal component of plant lignocellulose; it is a polysaccharide composed of glucose linked by β-1,4-bonds [1]. Among the organic compounds on earth, polysaccharides are the most abundant, and their decomposition into smaller fragments, such as monosaccharides and oligosaccharides is a necessary condition for effective utilization of polysaccharides [2]. Oligosaccharide is a linear or branched low-polymerization sugar composed of two to ten monosaccharide components connected by different glycosidic bonds. Researchers have found that oligosaccharides have a prebiotic effect, which not only promotes the growth of beneficial intestinal bacteria and affects the composition of the microbiota but also enhances the immune function of the body, playing an important role in physiological functions [3,4]. Cellulose degradation involves the coordination of multiple enzymes with different functions to degrade glucan into glucose. First, endoglucanase freely cuts the glycosidic bond at the nonreducing end of the amorphous region of the cellulose chain to produce oligomers of different lengths; then, exglucanase hydrolyzes cellobiose from the oligomers, and β-glucosidase hydrolyzes cellobiose to glucose. The three enzymes generally work together to complete the hydrolysis of cellulose [5]. Endo-β-1,4-glucanases (E.C. 3.2.1.4) have garnered considerable attention due to their ability to hydrolyze long cellulose chains into small molecular fragments. So far, cellulase has been employed in different industrial fields, such as improving the digestibility of low-quality feed and extracting oligosaccharides from agricultural by-products and agricultural residues for use as prebiotics [6,7].

In industrial applications, enzyme production is the most critical and costly step. In nature, numerous microorganisms produce endonucleases, such as fungi, bacteria, and actinomycetes [8]. Myxobacteria are gliding Gram-negative bacteria with multicellular behavior, characterized by the ability to degrade biological macromolecules [9,10,11]. Of the two groups of myxobacteria, one can lyse the living cells of other microorganisms, while the other can efficiently break down cellulose [11]. Because enzyme yield and activity of wild-type strains are often very low, the heterologous expression has become an important means of functional characterization, inexpensive production, and high-efficiency lignocellulose saccharification [12]. Endo-β-1,4-glucanases are present in at least 16 glycoside hydrolase families, of which GH6 is considered the most studied in terms of gene function and enzyme characterization [13]. GH6 endo-β-1,4-glucanases mainly exist in bacteria [14]. Until now, only a few GH6 endo-β-1,4-glucanases have exhibited heterologous expression.

We previously isolated and identified a strain of *Myxococcus* sp. B6-1 from the soil. In this study, a novel endo-β-1,4-glucanase gene from *Myxococcus* sp. B6-1 was cloned and expressed in *E. coli.* We further investigated the biochemical characteristics and straw saccharification ability of recombinant enzymes. To the best of our knowledge, this is the first report of GH6 cellulase from *Myxococcus* sp.

## 2. Materials and Methods

### 2.1. Chemicals, Strains, and Media

Glucose and other cello-oligosaccharide (G_2_-G_5_), carboxymethyl-cellulose (CMC), barley β-glucan, laminarin, xylan (from corncob), avicel, chitosan, and filter paper were obtained from McLean Biochemical Technology Co., Ltd. (Shanghai, China), whereas lichenin was purchased from Megazyme (Bray, Ireland). Other chemicals used in this study were provided by Sigma-Aldrich (Shanghai, China).

*Myxococcus* sp. B6-1 was isolated from soil and cultured in a lysogeny broth (LB). DH5 and BL21 (DE3) strains of *E. coli* from our laboratory were respectively utilized for gene cloning and expression. Plasmid pET-29a (Takara, Beijing, China) was used for expression.

### 2.2. Gene Cloning, Expression, and Purification of CelA257

The DNA of genomic organisms was isolated from *Myxococcus* sp. B6-1 and extracted using a previously described method [15]. The endoglucanase gene *CelA257* (GenBank registration number: MW248127) was amplified with the primers: *Cel*257F (5′- TAAGAAGGAGATATACATATGTGGCTCGCGGCCTCG-3′) and *Cel257*R (5′-GTGGTGGTGGTGGTGCTCGAGCGTACCGTCGATGAG-3′). PCR program included denaturation at 98 °C for 2 min, followed by 30 cycles: denaturation at 98 °C for 10 s, annealing at 65 °C for 5 s, polymerization at 72 °C for 10 s, with a final extension at 72 °C for 10 min. PCR products were purified, digested with *Nde*I and *Xho*I, and ligated into a pET-29a(+) vector to form pET-29a-*CelA257*. Following plasmid transformation into competent *E. coli* DH5α, positive transformants were isolated and sequenced. A verified recombinant plasmid was cultured on a LB medium containing kanamycin 50 mg/mL at 37 °C in *E. coli* BL21 (DE3). Isopropyl-β-D-thiogalactopyranoside (IPTG) solution (0.2 mM) was induced for protein overexpression until OD _600_ of strain reached 0.5–0.6. Following that, recombinant strain cells were gathered and lysed after being centrifuged at 8000× *g* for 20 min. After ultrasonic fragmentation (fragmentation conditions: 300 W, 70 times, ultrasound for 5 s, gap for 5 s, total time of 10 min), centrifugation was performed at 12,000 r/min and 4 °C for 15 min. The supernatant of recombinant CelA257 with a 6-His tag was purified with a Ni-NTA column and eluted with 20 mM Tris-HCl (pH 7.0) containing various imidazole concentrations (50–400 mM). Bradford’s method was used to determine the protein concentration [15], and the 10% SDS-PAGE was used to analyze the eluted protein.

### 2.3. Sequence Analysis and Homology Modeling of CelA257

Calculations were performed to determine the molecular mass and the isoelectric point of the compound using the Expasy tool (https://web.expasy.org, accessed on 23 July 2020). The protein sequence was analyzed using the NCBI database (http://blast.ncbi.nlm.nih.gov, accessed on 23 July 2020), and the sequence of amino acids analysis of CelA257 to other sequences was conducted by Clustal omega tools. Domains of structural and active sites were predicted using the Pfam database. Mega 5.0 Software and the Poisson model adjacency method were used to construct the phylogenetic tree. In addition, a 3D protein structural model of CelA257 was generated by the alpha fold2 online server (https://cryonet.ai/af2, accessed on 16 April 2021), and the structure of the active site was generated using Cel6 from *Mycobacterium tuberculosis* (PDB: 1UP3) as a template. Cel6 shared 42% identity and 60% query cover with the CelA257 sequence. Molecular graphics software PyMol was used to visualize the crystal 3D structure model.

### 2.4. Enzyme Assay

CMC (0.5%, *w*/*v*) was dissolved in 20 mM sodium phosphate buffer (pH 6.0) and incubated with enzyme for 1 h at 50 °C to determine enzyme activity The reducing sugar was determined according to the dinitrosalicylic acid (DNS) method [16]. For the substrate activity, 0.5% (*w*/*v*) barley β-glucan, lichenan, PASC, laminarin, avicel, filter paper, xylan, and chitosan were utilized. Triplicates of all experiments were performed. Chitosan substrate was prepared as previously described [17]. The amount of enzyme required to produce one μmol of reducing sugar per minute was defined as an enzyme unit. Chitosanase activity was determined using glucosamine as a standard, while other enzyme activities were determined using glucose as a standard.

### 2.5. Biochemical Properties of CelA257

CelA257 was tested in different buffers (with pH 3.0–10.5) to determine its optimal pH, including citrate buffer (pH 3.0, 4.0, 5.0, 6.0), sodium phosphate buffer (pH 6.0, 6.5, 7.0, 7.5), tris-HCl buffer (pH 7, 7.5, 8, 8.5, 9), and glycine-NaOH buffer (pH 9, 9.5, 10.0, 10.5). Various buffers (pH 3.0–10.5) were incubated at 4 °C for 24 h with aliquots of recombinant CelA257 in order to determine pH stability. The optimal reaction temperature was identified using a wide range of temperatures (4–100 °C) in pH 6.5 sodium phosphate buffer for 1 h. CelA257′s thermal stability was analyzed by depositing the solution in sodium phosphate buffer (pH 6.5) at 30–60 °C for 48 h. The impact of various metal ions (1 mM) and chemicals was measured in sodium phosphate buffer (pH 6.5). Reactants without any metal ions and chemicals were employed as controls, and the enzyme activity was considered 100%. To obtain the enzyme’s kinetic parameters, enzyme activity was determined using various CMC concentrations (1.2–4.0 mg/mL) in phosphate buffer (pH 6.5) at 50 °C for 1 h. The data were graphed using the Lineweaver–Burk method.

### 2.6. Hydrolytic Cello-Oligosaccharide Products Analysis

The hydrolysis mode of CelA257 was confirmed by hydrolyzing 1 mg/mL cello-oligosaccharides (G_2_-G_5_) in sodium phosphate buffer (pH 6.5) at 50 °C with 0.1 U CelA257. The samples were withdrawn after 6 and 12 h and boiled separately for 10 min. Following that, an analysis of the end products was conducted by thin layer liquid chromatography method (TLC). The final products of the reaction were applied onto silicone glass plates from Merck, Germany. These plates were then treated with a solvent system consisting of n-butanol, water, and acetic acid in a 2:1:1 *v*/*v*/*v* ratio. The solution was visualized by spraying with sulfuric/methanol acid (1:4, *v*/*v*) and heating at 85 °C for 15 min.

### 2.7. Agricultural Straw Saccharification

Different agricultural straws at 1% (*w*/*v*) were crushed through a 40-mesh sieve followed by blending in 20 mM PBS buffer (pH 6.5) containing 0 or 0.45 U crude CelA257 enzyme. Then the mixture was transferred to a 10 mL centrifuge tube, shaken at 50 °C for 48 h, and then boiled for 10 min, followed by centrifugation. The supernatants were utilized to determine the released reducing sugars of the reaction product using the DNS method. Finally, the number of milligrams of the reducing sugar released per milliliter of the reaction system was calculated.

## 3. Results and Discussion

### 3.1. Cloning and Analysis of CelA257 Sequence

**Figure 1:**
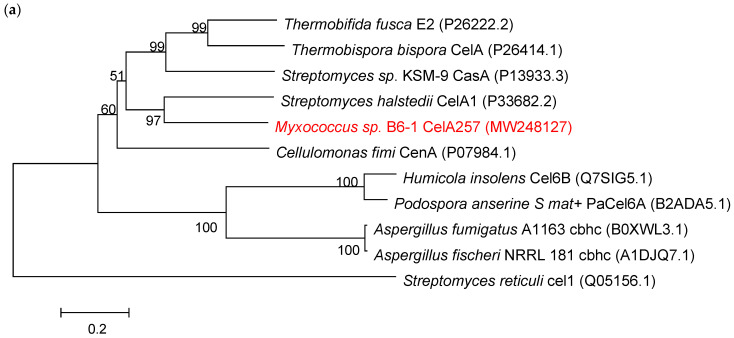

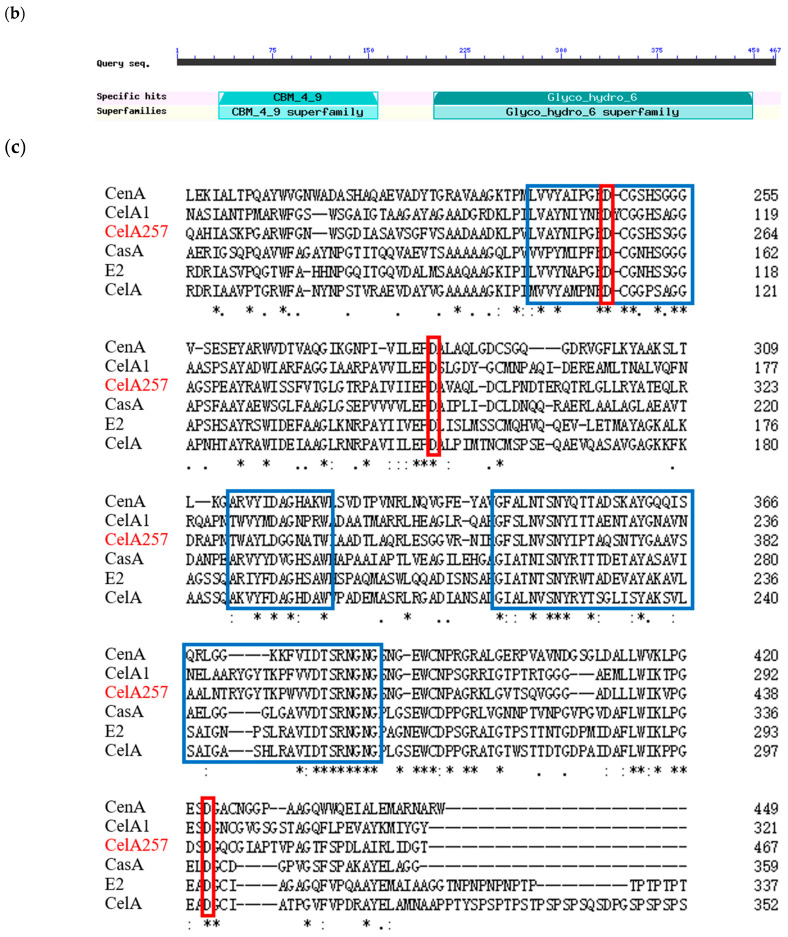
(**a**) The evolutionary relationships between CelA257 and other β-1,4-glucanases from various sources. (**b**) The structural domain prediction of CelA257. (**c**) Alignment comparison of amino acid sequences of CelA257 and other related β-1,4-glucanases. The red boxes denote active sites, and the blue boxes depict conserved amino acid sequences. An asterisk stands for a completely position of the residue.

Sequence alignment analysis of endo-β-1,4-glucanase gene *CelA257* revealed an open reading frame (ORF) of 1404 bp length that encoded 467 amino acids with the first 11 amino acids as a signal peptide sequence. Therefore, CelA257 must be an extracellular enzyme. The signal peptide was removed during heterologous expression. The theoretical pI was 5.01, and the predicted molecular mass was 48.7 kDa. Additionally, the phylogenic tree (Figure 1a) demonstrated that CelA257 was on the same branch as CelA1 from *Streptomyces halstedii* (NCBI Accession No. P33682.2). BLAST analysis manifested that CelA257 shared 54.7% of its identity with CelA1 from *Streptomyces halstedii* [18], followed by cusA from *Streptomyces* sp. KSM-9 (43.6%, P13933.3) [19], CenA from *Cellulomonas fimi* (43.3%, P07984.1) [20], E2 from *Thermobifida fusca* (40.8%, P26222.2) [21], and shared <40% of the sequence identity with other bacteria in GenBank database. The results indicated that CelA257 is a new GH6 endoglucanase. To effectively degrade cellulose, microorganisms typically produce a variety of endogenous and exogenous glycoside hydrolases (GH) [22]. Therefore, the carbohydrate active enzyme (CAZY) database classifies them functionally based on sequence similarity and structural properties [23]. These enzymes are classified into GH5, GH6, GH7, GH8, GH9, GH12, GH44, GH45, GH48, GH74, and GH124 families. However, despite the genetic and structural characterization of approximately 20 GH6 endo-b-1,4-glucanase genes and the availability of three crystal structures, reports on the biochemical and enzymatic properties of microbial endo-b-1,4-glucanases in the GH6 family are relatively limited [13].

CelA257 was predicted using the Pfam database to have structural domains from the CBM4-9 and GH6 families (Figure 1b). The amino acid sequence multiple alignment analysis showed three conserved sequences: 247–264 (region I), 329–340 (region II), and 358–405 (region III) (Figure 1c) [24]. Aspartic acid was reported to be a highly conserved amino acid residue required for GH6 enzyme catalytic activity, while Asp256, Asp294, and Asp441 were inferred as active sites of the endoglucanase from *Mycobacterium tuberculosis* [25].

Protein 3D modeling results revealed a narrow tunnel in the center of CelA257, where the active site was located (Figure S1c). CelA257 contains a typical (β/α) 8-barrel structure of the GH6 family (Figure S1d), which includes endoglucanase (EC 3.2.1.4) [25], cellobiohydrolase (EC 3.2.1.91) [26], and lichenase/endo-β-1,3-1,4-glucanase (EC 3.2.1.73) [14]. CelA257 active center was composed of three aspartic acid residues (Figure S1e,f), with Asp441 acting as the catalytic nucleophile and Asp294 as a proton donor [18]. The deduced N-terminal binding domain was analyzed as a member of the CBM4-9 family. The domain shared only 32.06% of its identity with cellulose-binding domain (CBD_N1_) from *Cellulomonas fimi* and comprised a typical jelly-roll-sandwich with two antiparallel β-sheets [27].

### 3.2. Expression and Purification of CelA257

**Figure 2:**
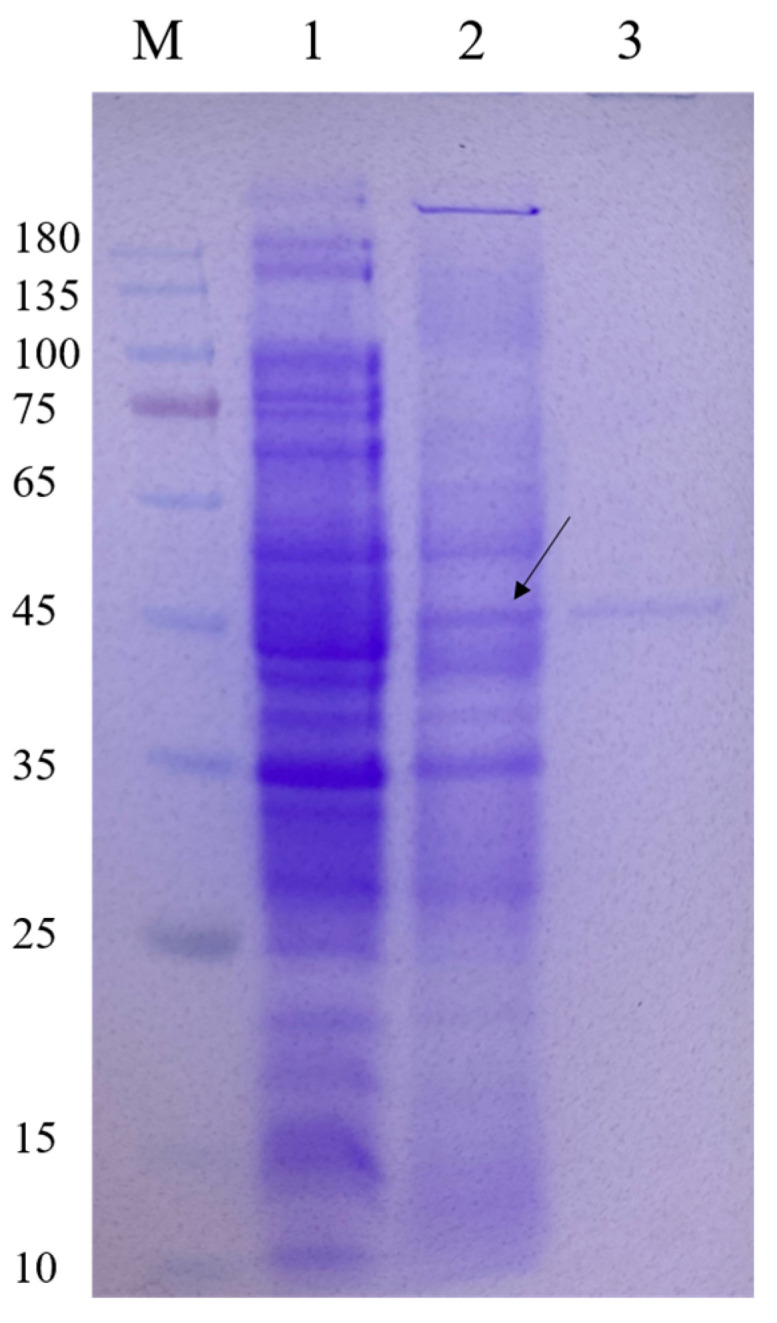
Expression and purification of CelA257. M: protein marker; lane 1: crude enzyme liquid of the supernatant of BL21 (DE3) cell lysate containing pET-29a; lane 2: crude enzyme liquid of the supernatant of recombinant strain cell lysate induced by IPTG; lane 3: purified CelA257. Arrow indicates the band where the target protein is located.

The *CelA257* gene was successfully expressed in *E. coli*. SDS-PAGE analysis of recombinant CelA257 soluble expression induced by IPTG was shown in the supernatant (Figure 2). Additionally, CelA257 was successfully purified using the Ni-NTA affinity column. After purification, CelA257 had a 16-fold increase in a specific activity (3.1 U/mg) with a 70.1% yield. We presumed that the low amount of recombinant enzyme production was due to the high G+C content (70.23%) in the DNA sequence. As a result, to obtain higher protein recovery, it is necessary to conduct codon optimization to improve gene expression in the following study. The molecular weight display result analyzed from SDS-PAGE (49 kDa) was in agreement with the predicted value (48.7 kDa). The molecular mass of CelA257 is slightly larger than PaCel6B (41.3 kDa) from *Podospora anserine* [28], smaller than CelL (56.0 kDa) from *Cellulosimicrobium funkei* HY-13 [13], and Cel6A (53.0 kDa) from *Cellulomonas pachnodae* [29]. These are both endo-β-1,4-glucanases containing a GH6 catalytic domain, whereas CelA257 comprises an additional CBM4-9 domain. Microbial glycoside hydrolases typically have a modular structure consisting of at least one catalytic domain (CD) connected to single or multiple carbohydrate-binding modules (CBMs) via a flexible connection region [30]. CBMs attach enzymes to the substrate surface, enhancing catalytic activity by increasing local enzyme concentration and potentially disrupting surface structure for more efficient catalysis, or targeting enzymes to specific substrates [31,32,33]. The binding of cellulase to cellulose is thought to be a step that limits cellulose hydrolysis, so CBMs are a key component of these modular cellulolytic proteins [34]. It was reported that CBM4-9 can bind to crystalline cellulose [35].

### 3.3. Characteristics of Recombinant CelA257

**Figure 3:**
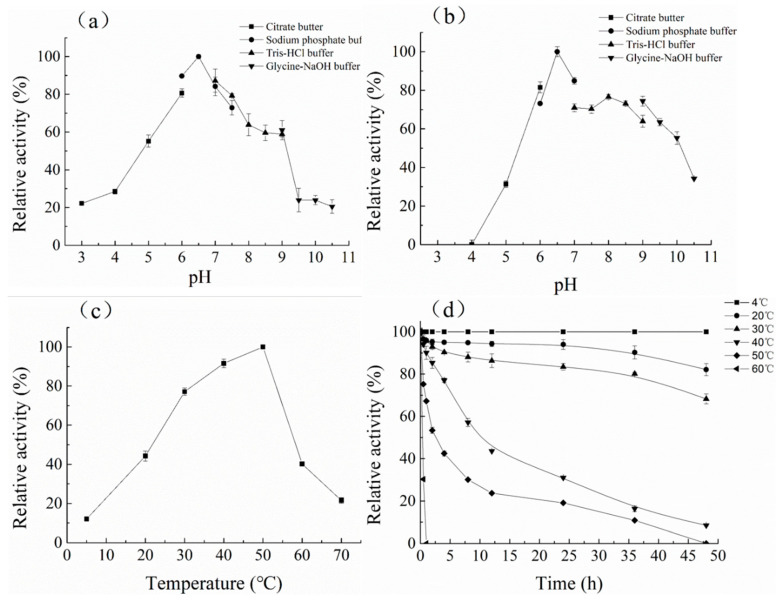
Optimum pH, temperature, and stability of CelA257: (**a**) optimal pH; (**b**) pH stability; (**c**) optimum temperature; (**d**) thermostability.

The optimum pH of CelA257 was 6.5 (Figure 3). The activity of CelA257 was greater than 50% at pH 5–9 and greater than 20% at pH 3 and 9.5. CelA257′s optimum pH was slightly higher than CelL (pH 5) from *Cellulosimicrobium funkei* HY-13 [13] and Cel6A (pH 5.5) from *C. pachnodae* [29], but lower than PaCel6B (pH 7–9) from *Podospora anserina* [28] and EG VI from *Humicola insolens* [36]. For pH stability, after 24 h incubation, the residual enzyme activity of CelA257 was more than 60% at pH 6–8. The optimum temperature (50 °C) was comparable to most GH6 endoglucanases. When incubated at 50 °C for 2 h, CelA257 remained at >50% activity, making it more thermostable than CelL (12 h). The main problems encountered by enzymes in commercial production and industrial applications include narrow pH ranges, inaccessibility of enzymes and substrates, thermal stability, end-product inhibition, and enzyme production costs [37]. After enzymatic characterization, CelA257 exerted a wide range of pH stability and temperature stability. These enzymatic properties provide favorable preconditions for industrial applications.

**Figure 4:**
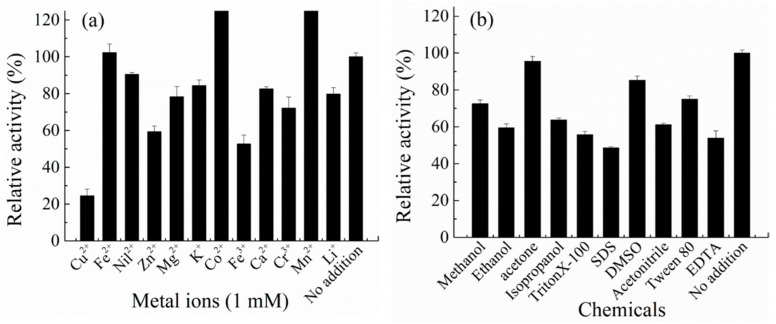
Effect of metal irons (**a**) and compounds (**b**) on the activity of CelA257.

CelA257 activity was raised by 1 mM Co^2+^ and Mn^2+^ to 128% and 131%, respectively (Figure 4). It was similar to CelL: the two metal ions both activated the enzyme activity slightly, while 5 mM Co^2+^ inhibited more than 50% of the activity of Cel6H-p35 and Cel6H-p23 from a compost metagenomic library [38]. Cu^2+^, Zn^2+^, and Fe^3+^ inhibited the activity to less than 60%, whereas the activity was only slightly inhibited by the other metal ions. In the presence of metal ions, the structure of the enzyme will change so that the activity of the enzyme will be activated or inhibited [39]. Therefore, in practical applications, the concentration of metal ions in the environment where the enzyme is added should be measured. In addition, most chemicals inhibited the activity to 50–80%, including 10% (*v*/*v*) methanol, acetonitrile, isopropanol, ethanol, 20 mg/mL tritonX-100, tween 80, 10 mM EDTA, and 2 mg/mL SDS. The residual activity remained more than 80% when 10% acetone and 5% DMSO were added.

### 3.4. Substrate Specificity of CelA257

CelA257 was relatively highly active against CMC (3.64 U/mg), barley β-glucan (17.58 U/mg), lichenan (6.49 U/mg), PASC (2.42 U/mg), and avicel (2.82 U/mg), but was inactive against chitosan (0.72 U/mg), laminarin (0.78 U/mg), and filter paper (0.54 U/mg) (Table 1). The results revealed that CelA257 exhibited broad activity against substrates with β-1,3-glucan linkages, β-1,4-glucan linkages, and mixed β-1,3-1,4-glucan linkages. CelA257 exerted efficient activity toward barley β-glucan, followed by lichenan, which contains mixed β-1,3-1,4-glucan linkages. The results are similar to those obtained with other GH6 endoglucanases; however, they are both endo-β-1,4-glucanases, not lichenases, because they hydrolyze only β-1,3-1,4-glucan linkages [40]. In addition, CelA257 exhibited chitosan and laminarin activity that was distinct from that of other GH6 endoglucanases. Insoluble cellulose avicel was degraded by CelA257, and the specific activity (2.8 U/mg) was higher than in CelL (0.6 U/mg) and Pacel6B (2.3 U/mg) (Table 2). It was reported that truncated Cel6D showed tiny activity to PASC and no activity to avicel; however, full-length Cel6D with CBM3 showed activity to avicel [41]. CBM 4-9 was confirmed to be necessary for crystalline cellulose hydrolysis of many microbial endo-β-1,4-glucanases [42]. We proposed that an additional carbohydrate-binding module of CelA257 can also promote the binding affinity to crystalline cellulose, hence increasing catalytic efficiency.

The enzyme kinetic parameter was analyzed by Lineweaver–Burk plots (see Figure S2). *K_m_* and *V_max_* values were 3.24 mg/mL and 4.74 U/mg toward CMC, respectively. *K_m_* value was higher than Cel6H-p35 (1.31 mg/mL) and Cel6H-p23 (1.32 mg/mL), but *V_max_* value was lower than Cel6H-p35 (8.75 U/mg) and Cel6H-p23 (6.57 U/mg) [38]. The higher *K_m_* value indicated that the affinity between the enzyme and the substrate was reduced, and the enzyme was not conducive to hydrolysis of the adjacent β-1,4-glucan linkages, resulting in the decrease in the *V_max_* values. The results suggested that CBM4-9 probably improved the binding to crystalline cellulose but not soluble cellulose, such as CMC.

### 3.5. Hydrolytic Cello-Oligosaccharide Products Analysis

**Figure 5:**
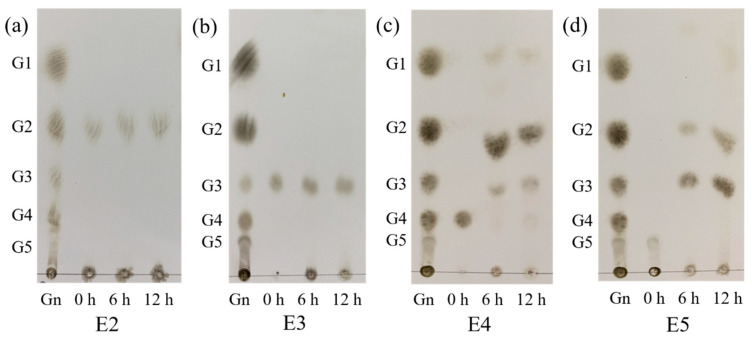
TLC analysis of CelA257 hydrolytic cello-oligosaccharide products (G_2_–G_5_) for 6 and 12 h, respectively. (**a**–**d**) stand for the products of CelA257 reaction with G_2_, G_3_, G_4_, and G_5_, respectively. E2, E3, E4, and E5 stand for the reaction samples of CelA257 with G_2_, G_3_, G_4_, and G_5_, respectively. (G_n_): Reference material including glucose (G_1_), cellobiose (G_2_), cellotriose (G_3_), cellotetraose (G_4_), and cellopentose (G_5_).

The model of CelA257 was analyzed by degrading cello-oligosaccharides, and the hydrolyzed products were analyzed by TLC (Figure 5). CelA257 hydrolyzed cello-oligosaccharides G_4_ and G_5_ rapidly at 6 h but did not hydrolyze G_2_ and G_3_ under reaction conditions. CelA257 hydrolyzed both G_4_ and G_5_ to G_2_ and G_3_. The results were similar to Cel6H-p35 from a compost metagenomic library. When Cel6H-p35 and CMC reacted for 12 or 24 h, Cel6H-p35 hydrolyzed cello-oligosaccharide G_5_ to G_2_ and G_3_ and G_4_ to G_2_ but hardly hydrolyzed G_3_ [38]. It was suggested that CelA257 and Cel6H-p35 exerted the same modes of hydrolysis and that CelA257 was an endo-type cellulase.

### 3.6. Agricultural Straw Saccharification

**Figure 6:**
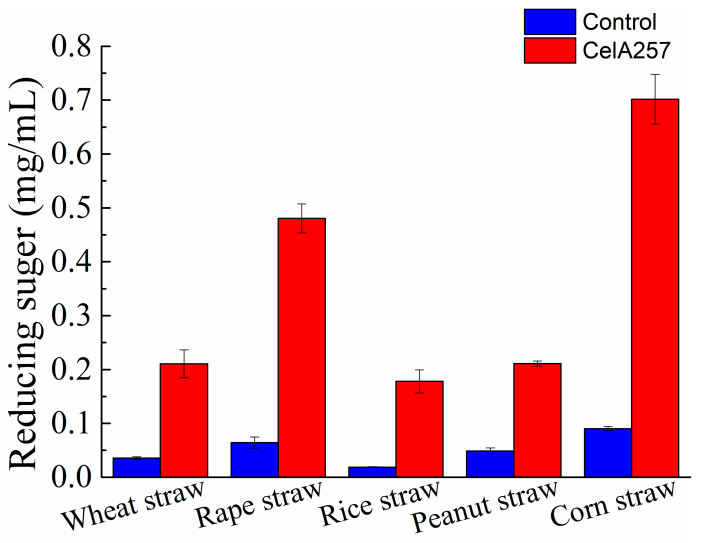
The yield of reducing sugars released from agricultural straws after hydrolysis by CelA257.

Five kinds of agricultural straw powers were used to analyze the CelA257′s saccharification effect. Compared with the control, the reducing sugars produced by treatment groups with CelA257 were significantly increased after 48 h. CelA257 improved the enzymolysis efficiency of all agricultural straws. The enhanced reducing sugar concentrations in wheat straw (0.18 mg/mL), rape straw (0.42 mg/mL), rice straw (0.16 mg/mL), peanut straw (0.16 mg/mL), and corn straw (0.61 mg/mL) are shown in Figure 6. The results are in accordance with the previous report, in which Cel-5A hydrolyzes unpretreated biomass raw materials [43]. In the industrial production of ethanol, endo-β-1,4-glucanase is an important enzyme to saccharify lignocellulosic biomass since it degrades long cellulose chains into shorter chains to facilitate subsequent hydrolysis. It is reported that using BGlc8H with extensive substrate specificity has improved the saccharification of pretreated reed and rice straw [44]. As a result, the saccharification effects of pretreated biomass with CelA257 will also be further investigated. For monogastric animals, beta-glucan is a substance that is difficult to digest, and adding cellulase can overcome the anti-nutritional factors of beta-glucan. With the further study of functional oligosaccharides, the effect of cello-oligosaccharides on the proliferation of lactic acid bacteria has been confirmed [45]. In addition, the effects of oligosaccharides on the physiological function of the body have also been more extensively studied and reported. In this experiment, the yield of oligosaccharides added with CelA257 to different straw powders was not further determined, and more in-depth studies will be conducted. Moreover, the synergistic effects of CelA257 with other endoglucanases, exoglucanases, and glucanases should be further investigated in the future. This study provides for a potential application of CelA257 as a pretreatment additive in biomass saccharification.

**Table 2:** Comparison of enzymatic properties between CelA257 and other GH6 β-1,4-glucanases.

Enzyme	Source	Molecular Size (kDa)	Optimal pH	Optimal Temperature	pH Stability	Thermal Stability				Specific Enzyme Activity (U/mg)						Reference	
							CMC	Barley β-Glucan	Lichenan	Chitosan	PASC	Xylan	Laminarin	Glucomannan	Avicel		
CelA257 CelL Cel6H-p35 Cel6H-p23	*Myxococcus* sp. B6-1 *Cellulosimicrobium funkei* HY-13 Compost metagenomic library Compost metagenomic library	48.7 56 35 23	6.5 5.0 5.5 5.5	50 °C 50 °C 50 °C 50 °C	6–9.5 4–10 - -	4–50 °C 4–50 °C 4–50 °C 4–50 °C	3.6 2.8 100 ^a^ 100 ^a^	17.6 6.2 328.8 334.5	6.5 3.0 62.7 82.9	0.7 - - -	2.4 1.9 - -	- - 17.9 4.3	0.8 - - -	- 2.5 - -	2.8 0.6 - -	This study [13] [32] [32]	
Pacel6B	*Podospora anserine*	41.3	7–9	45 °C	5–7	4–45 °C	5.5 ^b^	^c^	-	-	-	-		^c^	2.3	[28]	
EGVI Cel6A	*Humicola insolens* *C. pachnodae*	- 53	7.5 5.5	- 50–55 °C	- -	- -	- -	- -	- -	- -	- -	- -	- -	- -	- -	[29] [36]	
mgCel6A Thcel6A	Compost metagenome *Thermobifida halo-* *tolerans* YIM 90462	- 45.9	5.0 8.5	85 °C 55 °C	6–7 4–12	<70 °C 25–55 °C	^c^ ^c^	- ^c^	- ^-^	- -	^c^ -	- -	- -	^c^ -	^c^ ^c^	[46] [47]	
CfCel6C	*Cellulomonas fimi* ATCC484		5.5	65 °C	-	-	^c^	^c^	-	-	^c^	^c^	-	-	-	[48]	

- Not available. ^a,b^ Relative enzyme activity. ^c^ Lack of specific data.

## 4. Conclusions

In this study, a novel endo-β-1,4-glucanase gene (*CelA25*7) was cloned and expressed in *E. coli*. CelA257 was stable at temperatures ranging from 4 to 50 °C and pH ranging from 6 to 9.5. The results of oligosaccharide hydrolysis indicated that CelA257 is an endo-type cellulase. CelA257 exerted broad substrate specificity and high activity to crystalline cellulose. CelA257 was added to agricultural straws to increase their saccharification efficiency. These findings suggested that it may be used as a biomass saccharification supplement.

## Figures and Tables

**Table 1:** Substrate specificity of CelA257.

Substrate	Specific Activity (U/mg)
CMC	3.64 ± 0.03
Chitosan	0.72 ± 0.02
laminarin	0.78 ± 0.05
Barley β-glucan	17.58 ± 0.57
Lichenan	6.49 ± 0.26
Filter paper	0.54 ± 0.01
PASC	2.42 ± 0.01
Avicel	2.82 ± 0.07

## Data Availability

Data is contained within the article or supplementary material.

## Supplementary Materials

The following supporting information can be downloaded at: https://www.mdpi.com/article/10.3390/foods12132517/s1, Figure S1: Three-dimensional structural model of CelA257 protein. Figure S2: Lineweaver–Burk plots of CelA257 using CMC as substrate.
