# Supplementary material for: Biochemical Characterization of Novel GH6 Endoglucanase from Myxococcus sp. B6-1 and Its Effects on Agricultural Straws Saccharification
Source: Foods. 2023 Jun 28;12(13):2517. doi: 10.3390/foods12132517 (PMC10340332; doi:10.3390/foods12132517)

Figure S1. Three-dimensional structural model of CelA257 protein. (**a**) Surface display of CelA257 protein. (**b**) Ribbon display of CelA257 protein. (**c**) Surface display of catalytic domain. (**d**) Ribbon display of catalytic domain. (**e**) The active location in the catalytic domain's cleft. The ribbon in green stands for CelA257, and the ribbon in cyan stands for 1UP3. (**f**) The structure of amino acid residues in the active site. Red sticks are used to stand for the amino residues of CelA257, while blue sticks stand for the amino residues of 1UP3.

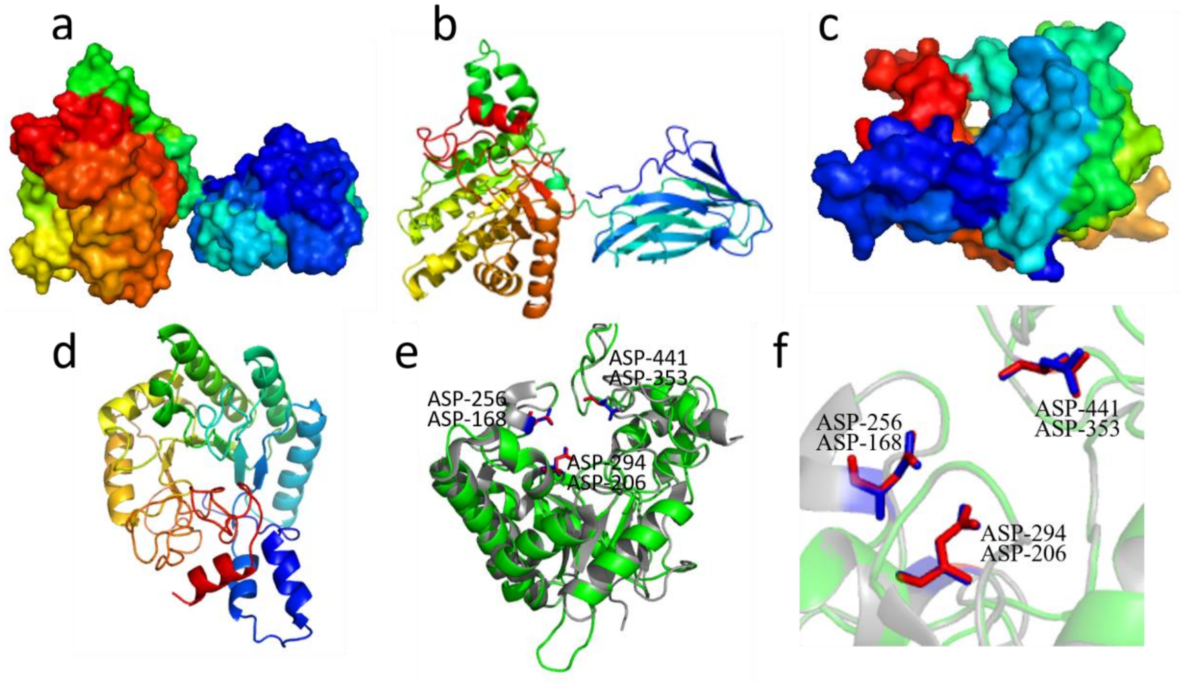

Figure S2. Lineweaver-Burk plots of CelA257 using CMC as substrate.

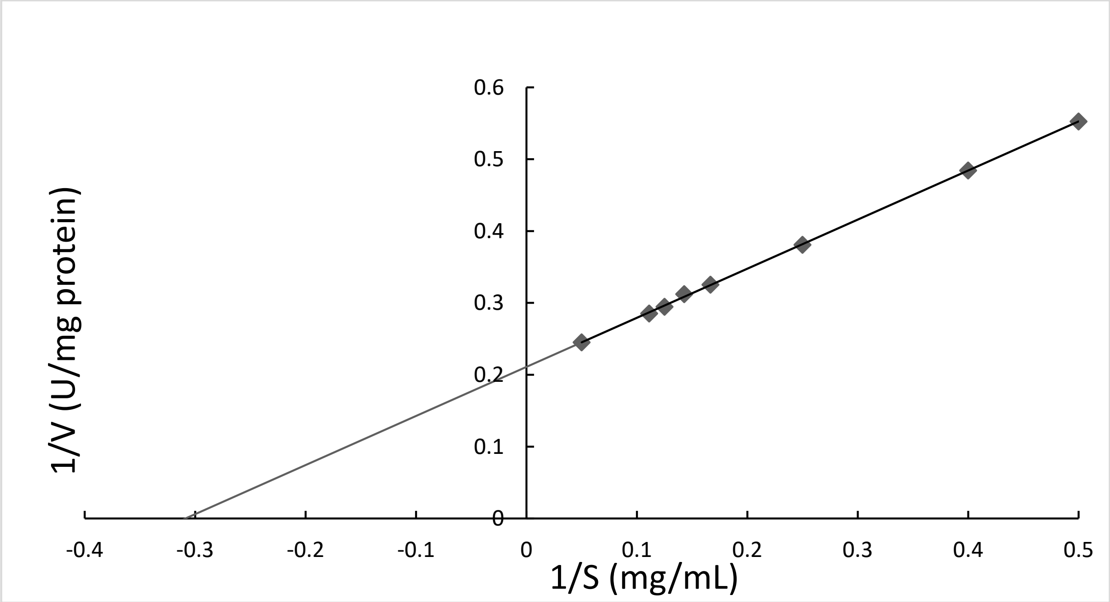

Supplement: Supplementary file 1 [file foods-12-02517-s001.zip › foods-2442942-supplementary.pdf]
